# Natural Killer Cells in Myeloid Malignancies: Immune Surveillance, NK Cell Dysfunction, and Pharmacological Opportunities to Bolster the Endogenous NK Cells

**DOI:** 10.3389/fimmu.2019.02357

**Published:** 2019-10-11

**Authors:** Mattias Carlsten, Marcus Järås

**Affiliations:** ^1^Department of Medicine, Huddinge, Center for Hematology and Regenerative Medicine, Karolinska Institutet, Stockholm, Sweden; ^2^Division of Clinical Genetics, Department of Laboratory Medicine, Lund University, Lund, Sweden

**Keywords:** NK cells, myeloid malignancy, cancer immunotherapy, drug development, NK cell dysfunction

## Abstract

Natural killer (NK) cells are large granular lymphocytes involved in our defense against certain virus-infected and malignant cells. In contrast to T cells, NK cells elicit rapid anti-tumor responses based on signals from activating and inhibitory cell surface receptors. They also lyse target cells via antibody-dependent cellular cytotoxicity, a critical mode of action of several therapeutic antibodies used to treat cancer. A body of evidence shows that NK cells can exhibit potent anti-tumor activity against chronic myeloid leukemia (CML), acute myeloid leukemia (AML), and myelodysplastic syndromes (MDS). However, disease-associated mechanisms often restrain the proper functions of endogenous NK cells, leading to inadequate tumor control and risk for disease progression. Although allogeneic NK cells can prevent leukemia relapse in certain settings of stem cell transplantation, not all patients are eligible for this type of therapy. Moreover, remissions induced by adoptively infused NK cells are only transient and require subsequent therapy to maintain durable responses. Hence, new strategies are needed to trigger full and durable anti-leukemia responses by NK cells in patients with myeloid malignancies. To achieve this, we need to better understand the interplay between the malignant cells, their microenvironment, and the NK cells. This review focuses on mechanisms that are involved in suppressing NK cells in patients with myeloid leukemia and MDS, and means to restore their full anti-tumor potential. It also discusses novel molecular targets and approaches, such as bi- and tri-specific antibodies and immune checkpoint inhibitors, to redirect and/or unleash the NK cells against the leukemic cells.

## Introduction to Natural Killer Cells, Their Receptors, and Role in the Immune System

The natural killer (NK) cell was discovered in the mid-1970s based on its ability to lyse certain tumor cells without prior sensitization of the host (1–4). Based on this, and the understanding that both T and B cells in contrast to NK cells need to undergo somatic gene rearrangement to become fully functional with specific immunity that quickly respond upon recalling, NK cells have for long been considered innate immune cells. However, more recent data have challenged this perception by demonstrating that NK cells also can carry memory-like features (5). Today, NK cells are explored in a wide variety of contexts, including, but not limited to, infectious diseases, autoimmunity, pregnancy, and cancer. Thus, from an unknown cell type with undetermined biological meaning and significance in the mid-1970s, it has now more than 40 years later been recognized that NK cells are key components of our immune system.

**Figure 1:**
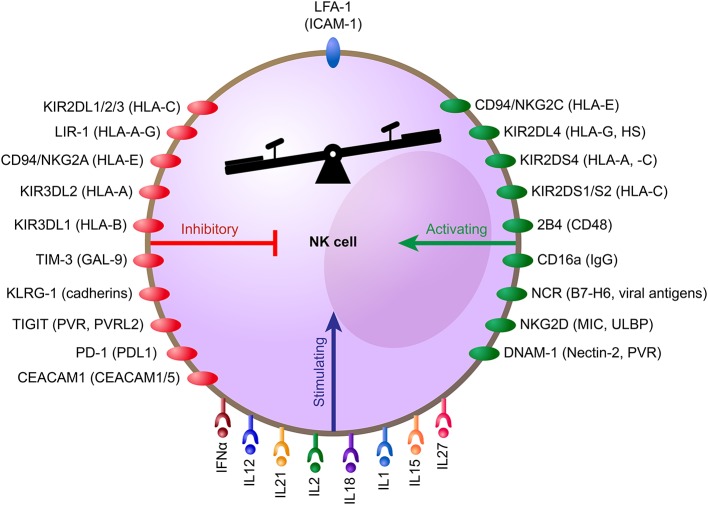
NK cell receptors, their function, and ligands. Schematic illustration showing how NK cell activity and cytotoxicity are controlled by signals from cell surface receptors. Cytokines and corresponding cytokine receptors on the NK cell are shown at the lower part of the NK cell. Inhibitory signals triggered by receptors (red) upon engagement of their ligands (in brackets) are shown on the left side of the NK cell. Activating signals triggered by receptors (green) upon engagement of their ligands (in brackets) are shown on the right side. Binding of LFA-1 (blue) on NK cells to ICAM-1 on target cells direct the granulae release toward the target cell, which is needed for efficient target cell lysis.

NK cells have traditionally been classified as group 1 innate lymphoid cells and develop from hematopoietic stem cells (HSCs) while maturing outside the bone marrow compartment (6, 7). They have the capability to migrate to a number of tissues to launch immune responses to infections and cancer (8). The basis for target recognition by NK cells was revealed in the mid-1980s when the “missing-self” hypothesis was postulated (9). However, as predicted by the investigators at that time, activation signals are needed in addition to “missing-self” to trigger cytotoxicity (10). Today, we know that a delicate interplay between an array of germ-line encoded receptors expressed on the NK cell surface control NK cell degranulation (Figure 1) (11, 12), a cytotoxicity mechanism that lyses target cells via the release of substances such as perforin and granzymes. The key receptors controlling self-recognition by human NK cells are HLA class I-binding receptors, including the Killer Immunoglobulin-like Receptor (KIR) family as well as the Natural Killer Group 2A (NKG2A) and Leukocyte immunoglobulin-like receptor subfamily B member 1 (LILRB1, also referred to as LIR-1) (11). The inhibitory KIRs and the NKG2A receptor have also been shown to be involved in NK cell education, a functional maturation process that allows self-inhibited NK cells to become potent killers upon interaction with cells losing self-HLA class I expression (13). In contrast to the inhibitory receptors, an array of activation, co-activation, and adhesion receptors such as the natural cytotoxicity receptors (NCRs) NKp30 and NKp46 and the NKG2D, 2B4, and DNAM-1 receptors trigger NK cell activation following binding to ligands up-regulated on cells undergoing stress and/or infection (11). Under normal conditions when NK cells are not heavily activated by cytokines, at least two of these receptors need to be stimulated simultaneously to trigger degranulation (14). This is in contrast to the FcγRIIIA receptor (CD16a), that upon ligation to the Fc portion of an antibody bound to a target cell alone potently can trigger degranulation (15). This process is referred to as antibody-dependent cellular cytotoxicity (ADCC). Importantly, engagement of the LFA-1 receptor on the NK cell is required in most situations to direct the granulae release toward the target cell and thereby trigger efficient target lysis (15). The latter adds another layer to how NK cell cytotoxicity is regulated. In addition to target cell lysis via the release of granzymes and perforin, NK cells also kill cells via stimulation of death receptors on the target cell surface, which triggers caspase-dependent apoptosis (16). Both TNF-related apoptosis-inducing ligand (TRAIL) and Fas ligand (FasL) on the NK cell surface can trigger caspase-mediated apoptosis in target cells expressing TRAIL-R1 and/or -R2 and Fas, respectively (17). Importantly, NK cells do not only kill infected and tumor-transformed cells via these mechanisms, but also utilize these receptors to control immune responses by killing, i.e., T cells (18, 19).

NK cells have several functions in the immune system. Based on data from individuals with severe NK cell deficiencies and data from experimental animal models, it has been recognized that they are highly implicated in controlling Epstein–Barr virus (EBV) (20–22), but also involved in the defense against Herpes simplex virus (HSV) infections (23). Moreover, it is well-established that NK cells can react to cytomegalovirus (CMV) infection and prevent CMV reactivation following allogeneic stem cell transplantation (SCT) (24, 25). Beyond their role in viral infections, NK cells have an immunomodulatory role either by directly controlling other immune cells (18, 19) or by release of chemokines and cytokines that can attract and stimulate both innate and adaptive components of the immune system (26, 27). NK cells also have a documented role in pregnancy (28). Given the rapid advances in our understanding of NK cells, additional functions for these cells in the body will likely be unveiled in the near future.

The role for NK cells in cancer has been addressed since the discovery of this lymphocyte subset. Over the years, it has become clear that NK cells are involved in tumor immune surveillance (29). Indirect evidence comes from cohort studies showing that individuals with poor NK cell function early in life have a higher risk of presenting with cancer compared to matched controls (30). Clinical observations also indicate that a ligand repertoire on acute myeloid leukemia (AML) blasts favoring NK cell activation is positively linked to better outcome of patients undergoing chemotherapy (31). More direct evidence from animal models indicate that knock-out of key NK cell receptors such as NKG2D and DNAM-1 leads to higher incidence of tumor formation compared to in mice with wild-type expression of these receptors (32, 33). Another line of evidence comes from clinical studies on allogeneic SCT and adoptive NK cell infusion showing NK cells can be utilized to treat patients with cancers, including myeloid malignancies (34, 35). This has opened up a new field focusing on NK cell-based cancer immunotherapies that all aim to bolster the NK cell tumor targeting capacity to improve outcomes of patients with cancer (36). In parallel to this development, more and more studies also demonstrate that NK cells in patients with cancer are defective, and in some cases also few in numbers, indicating a potential breach of NK cell-mediated tumor immune surveillance that may facilitate disease progression. Dysfunctional NK cells have been reported in both solid tumors (37) and hematological malignancies, including myeloid malignancies (38, 39). For some of these cancers, it has also been proposed that restoration of the NK cell function after treatment with cytoreductive chemotherapy, or other targeted drugs, can re-establish NK cell-mediated cancer control. As will be discussed below, a prime example of this is chronic myeloid leukemia (CML), but there are also data reporting that this can occur in other myeloid malignancies such as AML and myelodysplastic syndromes (MDS) as well as in chronic myelomonocytic leukemia (CMML). Notably, in contrast to malignancies of the myeloid lineage, data on the role for NK cells in targeting B cell-derived leukemias such as acute lymphoblastic leukemia (ALL) and chronic lymphocytic leukemia (CLL) are less clear and will not be discussed in this review.

This review will focus on our current understanding of the role for NK cells in targeting malignant myeloid cells and thereby preventing the initiation and/or the progression of AML, MDS, and CML, and how malignant cells in these diseases can evade NK cell recognition. Methods to circumvent and/or restore this imbalance will be discussed. In the emerging era of immune checkpoint inhibitors and tumor targeting antibodies, including bi- and tri-specific killer engagers, the review will have a special focus on the mechanisms governing suppressed NK cell function in these diseases and means to restore the NK cell phenotype and function to define potential opportunities to use such drugs in clinical practice. As other reviews and articles have comprehensively covered the role of NK cells in settings of allogeneic SCT and adoptive cell transfer to treat AML, CML, or MDS, our review will only touch upon these topics. Instead, this review will have a particular focus on the endogenous NK cells and their therapeutic potential and limitations.

## Evidence for NK Cell-Mediated Targeting of Malignant Myeloid Cells and Data Supporting a Role for NK Cells in the Treatment and Control of CML, AML, and MDS

### Introduction to CML, AML, and MDS—Biological and Clinical Similarities and Differences

Although originating from the myeloid lineage, CML with its 9;22 translocation that creates the BCR/ABL fusion gene is biologically and clinically very different from AML and MDS. From being a disease with high mortality following transformation to blast crisis, where allogeneic SCT was considered the only treatment option that could offer a potential cure, CML is now efficiently treated using tyrosine kinase inhibitors (TKIs) (40). Unfortunately, similar approaches have not been equally successful for MDS and AML, diseases that do not express the BCR/ABL tyrosine kinase fusion gene but are rather triggered and driven by multiple mutations. In contrast to CML, the more aggressive AML disease as well as high-risk MDS are generally associated with dismal outcome. Hence, there is an urgent need of identifying new and more efficient treatment options for these malignancies. To successfully design new therapies that induce durable responses, it is likely key to understand the underlying disease and how it potentially compromises the immune system. For deeper understanding of the CML, AML, and MDS diseases *per se*, please see references (41–43).

### NK Cell-Mediated Targeting of Tumor-Transformed Myeloid Cells via Natural Cytotoxicity and Its Role in Treating Patients With Myeloid Malignancies

Preclinical studies have firmly demonstrated that NK cells can kill leukemic cells of the myeloid lineage. Data derive from studies using leukemia cell lines, but also freshly isolated leukemic blasts from patients with CML, AML, or MDS. In addition to these studies exploring the potential of primary human NK cells, studies have also demonstrated that NK cell lines can target primary as well as immortalized AML and CML cells (44).

The first series of experimental studies on this topic were conducted using AML and CML blasts and published just a few years after the NK cell was first described. In a small *ex vivo* study published already 1983, investigators were able to show that freshly explanted CML blasts could be lysed by interferon (IFN)-activated NK cells from healthy donors (45). As demonstrated in a paper from the group of Ronald Herberman a few years later (1989), the main basis for prevention of clonogenic growth of freshly explanted AML and CML blasts or cells from pre-leukemic patients (today called MDS) was cell-to-cell interaction, although soluble factors produced by the NK cells were also involved (46). Importantly, the anti-leukemia activity was only detectable in these experiments when enriched NK cell populations were used. The need for cell-to-cell contact to trigger NK cell-mediated inhibition of autologous CML blast growth has later been verified in other studies (47).

The more recent studies on this topic have mainly focused on targeting AML cells with NK cells *in vitro*. Most studies have addressed the potential of resting and overnight cytokine-activated [i.e., interleukin (IL)-2 or IFN] NK cells (39, 45). Other studies have explored the potential of *ex vivo* expanded NK cells (48, 49). The molecular specificity of NK cell-mediated cytotoxicity of leukemic cells is based on several receptor–ligand interactions. For instance, the NKG2D and DNAM-1 receptors as well as the NCRs have been reported important for the targeting of AML and CML blasts (50–52), whereas studies on freshly isolated MDS blasts have revealed that the DNAM-1 receptor is central with contributions from the NKG2D receptor and the NCRs NKp30 and NKp46 (39). It is also evident from the literature that blockade of inhibitory KIR, CD94/NKG2A, and LIR-1 augment NK cell-mediated killing of leukemic blasts (53), indicating that they express enough HLA class I to at least partially inhibit NK cells. The role for these activation and inhibition receptors in targeting of myeloid malignancies by NK cells will be discussed in more detail in section Means to Restore NK Cell Function and Trigger Their Cytotoxicity Against Myeloid Malignancies below.

### Exploring Human NK Cells to Target CML, AML, and MDS Cells Implanted in Animal Models

Until today, the vast majority of xenografted mouse models used to explore the anti-leukemia potential of primary human NK cells have focused on human leukemia cell lines. One of the major reasons for this is that engraftment of primary AML, CML, and MDS cells has historically been difficult, with only recently reaching robust and reliable engraftment rates in optimized models (54–56). Furthermore, the use of human leukemia cell lines enables the researcher to introduce luciferase and/or fluorescent proteins (such as green fluorescent protein; GFP) to efficiently track the tumor burden in the mice. This is exemplified in several studies on human xenografted leukemia, which will be discussed below.

*Ex vivo* expanded peripheral blood NK cells can prevent leukemia development in severe combined immunodeficiency disease (SCID)-beige mice and NOD-*scid* IL2Rgamma^null^ (NSG) mice inoculated with K562 cells (49, 57). In line with this, investigators have also shown that NK cells generated from CD34^+^ hematopoietic stem cells *ex vivo* as well as from cord blood cells can clear K562 cells in mice (58, 59). Moreover, cytokine-induced killer cells, featuring a mixed NK and T-cell phenotype, were capable of mediating potent reduction of tumor burden in mice engrafted with the AML cell line THP-1 (60). In contrast to utilizing human leukemia cell lines as targets in the animal models, the ability of primary human NK cells to target xenografted primary myeloid leukemia in mice has only been highlighted in few studies. One example of the latter comes from a study that efficiently utilized *ex vivo* expanded human NK cells expressing a single KIR (61). There are also data addressing the role for primary human NK cells targeting primary xenografted autologous myeloid leukemia. As demonstrated by Siegler et al. (62), *ex vivo* expanded NK cells are able to target xenografted autologous AML blasts. In this study, the authors speculate that up-regulation of the NKG2D receptor and the NCRs following *ex vivo* expansion and activation of the NK cells prior to adoptive infusion into the mice was key to govern the anti-leukemic effects. Although several models have been used to establish that primary human NK cells can target leukemic cells implanted in mice, we predict that development of more advanced models will be valuable tools to explore how the leukemic cells can negatively affect the adoptively infused NK cells in detail.

### Data on Utilizing NK Cells to Treat Patients With Myeloid Malignancies

Data supporting NK cell-mediated rejection and control of myeloid leukemia in patients have been generated from studies on allogeneic SCT. In 2002, Ruggeri et al. reported that KIR-ligand mismatching in the graft-vs.-host (GvH) direction of donor NK cells was key to prevent AML relapse following haplo-identical SCT (34). In line with these data, Hsu et al. also demonstrated that the genomic lack of one or more ligands in the recipient for donor KIR was associated with improved outcome in AML and MDS in settings of T-cell-depleted HLA-identical sibling transplantations (63). Studies on large transplantation cohorts have also linked certain KIR genotypes and KIR–KIR-ligand genotype pairs that also include activating KIRs to post-transplant control of leukemia (64–66). More recent data also indicate the expansion of adaptive NK cell subsets post-transplantation is linked to improved outcome in AML, which adds an additional layer to the role of NK cells in post-transplant relapse protection (67).

The potential of utilizing mature NK cells in setting of adoptive cell transfer to treat myeloid leukemia patients was demonstrated by Miller et al. (35). In this study, 19 patients with relapsed/refractory AML were treated with overnight IL-2-activated haplo-identical NK cells. In this patient population with very advanced high-risk disease, 5 out of 19 patients had a complete remission (CR). Remarkably, four out of the five responders had received donor NK cells with a KIR-ligand mismatch in the GvH direction. Following this publication, there has been an explosion of clinical trials demonstrating improved outcome of AML and MDS patients treated with NK cells in different settings (49, 68–73). Of note, most of these studies have not been able to demonstrate a beneficial effect of KIR-ligand mismatching. This may relate to the relative loss of cell surface HLA class I expression on the myeloid blasts compared to the lymphocytes. As demonstrated by Verheyden et al., the relative expression of HLA class I, and especially HLA-C, was markedly down-regulated on myeloid blasts compared to autologous T cells potentially leading to reduced inhibition by HLA-Bw4- and HLA-C-binding KIRs and thereby attenuation of the role for KIR-ligand mismatching (74). Instead, data indicate that outcomes following adoptive NK cell therapy are positively predicted by presence and expansion of donor NK cells and dampened host immune activation post NK cell infusion as well as removal of regulatory T cells prior to NK cell infusion (72, 75). As shown by Romee et al., adoptive infusion of memory-like NK cells can trigger anti-AML responses while leading to improved persistence of the NK cells (49). Another factor that has been highlighted in more recent studies is the dose of alloreactive NK cells. This has been demonstrated in the setting of adoptive NK cell infusion as post-consolidation therapy for elderly patients with AML (70), and also in the setting of pre-allogeneic SCT for patients with AML, MDS, or CML (71). Nevertheless, due to the relatively poor persistence of adoptively infused NK cells, objective clinical responses induced in these settings are only transient. Hence, these protocols can be used as a bridge to an allogeneic SCT or maybe to deepen responses in the post-consolidation setting, but not cure patients with myeloid malignancies.

Collectively, the capacity of NK cells to target AML, MDS, and CML blasts *in vitro* and in xenografted mouse models is well-documented with clear involvements of the NKG2D and DNAM-1 receptors, but also the NCRs. Based on data from CML, AML, and MDS patients undergoing allogeneic SCT, it is clear that NK cells do have a role in the clearance and control of myeloid malignancies in certain settings. Although adoptive NK cell transfer can be effective and adds to the notion that NK cells can be utilized to target myeloid malignancies, clinical remissions are only transient. An alternative approach that may induce durable remissions without the need of cellular therapy would be to bolster the anti-tumor potential of the endogenous NK cells. This approach has until now been relatively unexplored and likely been limited due to leukemia-induced dysfunction of the NK cells in these patients. With the increased knowledge, we predict that this approach will be a more viable option in the near future. NK cell dysfunction in myeloid malignancies and how to restore it will be described in the following sections of this review.

## NK Cell Function and Maturation in Patients With Myeloid Malignancies at Diagnosis and Upon Treatment

### NK Cell Numbers and Function During Treatment and Disease Progression

The anti-leukemic activity of NK cells inversely correlates to disease progression in AML—the NK cells are suppressed at diagnosis, restored at remission, and again suppressed at relapse (76, 77). Similarly, in MDS, the cytolytic activity of NK cells is severely altered, even in the presence of IL-2 stimulation *in vitro*, as compared to NK cells from healthy donors (78). In CML, the NK cells decrease in number along disease progression, respond less to stimuli, and exhibit reduced cytolytic activity (79, 80). Similar to AML patients in CR, CML patients with a major molecular response (MMR) to TKIs have restored cytolytic functions of NK cells (81). In support of NK cells being involved in immune control of CML cells, patients with a high percentage of NK cells at the time of TKI discontinuation had a better long-term outcome (82). Also, the role for “missing-self” reactivity by endogenous uneducated NK cells has been highlighted in CML patients treated with TKI. Patients carrying non-interacting KIR3DL1 and HLA-B allele pairs, leading to less inhibition of NK cells upon interaction with CML blasts, have better outcome upon TKI treatment (83). In AML, higher cytolytic activity of NK cells predicts a better long-term outcome of patients at both diagnosis and in remission (84–87). In addition, high expression of the activating NK cell receptors NKp30 or NKp46 predicts a better outcome (38, 88–90). The role for “missing-self” genotypes has, like for CML, also been associated with an improved outcome in AML following post-consolidation treatment with dihydrochloride and low-dose IL-2 that activates NK cells (91). In a follow-up study, the investigators identified that the efficacy against AML was linked to a dimorphism in HLA-B at amino acid −21 that has an impact on NK cell education (92), again supporting a critical role for NK cells in this disease. In a separate study, the outcome following treatment of AML and high-risk MDS with the hypomethylating agent Azacytidine could be predicted by NK cell function after three to six cycles (93). Taken together, NK cell function is often suppressed upon diagnosis and at disease progression of myeloid malignancies, but restored in remission. Increased number of NK cells as well as more activated NK cells at diagnosis and following remission correlate with better outcome for patients treated with hypomethylating agents, TKI and IL-2. These findings suggest that NK cells have a central role in the control of myeloid malignancies by counteracting disease progression.

### Altered Maturation of NK Cells in Myeloid Malignancies

Normal NK cell differentiation is defined by combinations of markers that include CD56, CD16a, CD57, KIRs, and NKG2A (94). Immature NK cells (CD56^bright^CD16a^−^CD57^−^) are cytokine-producing cells with low cytotoxic activity, whereas more mature NK cells (CD56^dim^CD16a^+^CD57^+^) have higher cytotoxic activity (95). NK cell differentiation is characterized by down-regulation of NKG2A and up-regulation of KIR, which alter their reactivity given the HLA class I repertoire expressed on the target cell. In myeloid malignancies, NK cell maturation was suggested to be perturbed with a selective loss of an immature NK cell population in both AML patients and in leukemic mice (77, 96). This loss of primitive NK cells was accompanied by an increased percentage of phenotypically more mature (CD56^dim^KIR^+^CD57^+^) NK cells in the peripheral blood of AML patients (97). However, opposing findings of a decreased proportion of mature NK cells (CD56^dim^CD16a/CD57^bright^) in AML and MDS have also been reported (98). Consistent with previous findings by Martner et al. (99), Chretien et al. divided the AML patients into three subtypes based on the NK cell maturation and found that patients with more immature NK cells had reduced relapse free and overall survival, suggesting that disease-induced alterations in NK cell maturation affect patient outcome (100). Therapies can also affect the differentiation stage of the NK cells. In first remission, an increased percentage of immature (CD56^bright^) NK cells in AML patients has been observed, possibly because the NK cells are under reconstitution after intense chemotherapy (101). In CML, treatment with the TKI dasatinib is associated with differentiation of NK cells (102). Upon MMR or molecular response (MR), CML patients have more mature cytolytic NK cells (CD57^+^CD62L^−^), indicating restoration of NK cell function (81). Although several of the studies described above found that disease-induced mechanisms and certain treatments influence the maturation of NK cells in myeloid malignancies, interpretations of how the maturation stage of NK cells in this context affect their anti-leukemic activity is so far mainly based on correlative findings. Hence, more studies are needed to clarify how the maturation stage of NK cells in myeloid malignancies is perturbed and affected by treatment both in a short- and, more importantly, long-term perspective. Single-cell RNA-sequencing, which is an emerging methodology that recently has increased our understanding of NK cell regulation (103, 104), has the potential to further clarify how NK cell maturation is affected by treatment.

Collectively, disease-induced mechanisms in myeloid malignancies negatively affect core properties of NK cells such as their differentiation and cytotoxic potential correlating to disease progression. Moreover, the NK cell function during and after treatment is linked to treatment responses and outcome, suggesting that NK cells play a key role in controlling myeloid malignancies. By further characterizing the mechanistic basis for how NK cell dysfunctions arise and how NK cell differentiation and function is modulated by treatment may translate into new treatment opportunities for myeloid malignancies as discussed in more detail below.

## The Impact of Shared Genetic Aberrations Between NK Cells and Malignant Myeloid Cells

**Figure 2:**
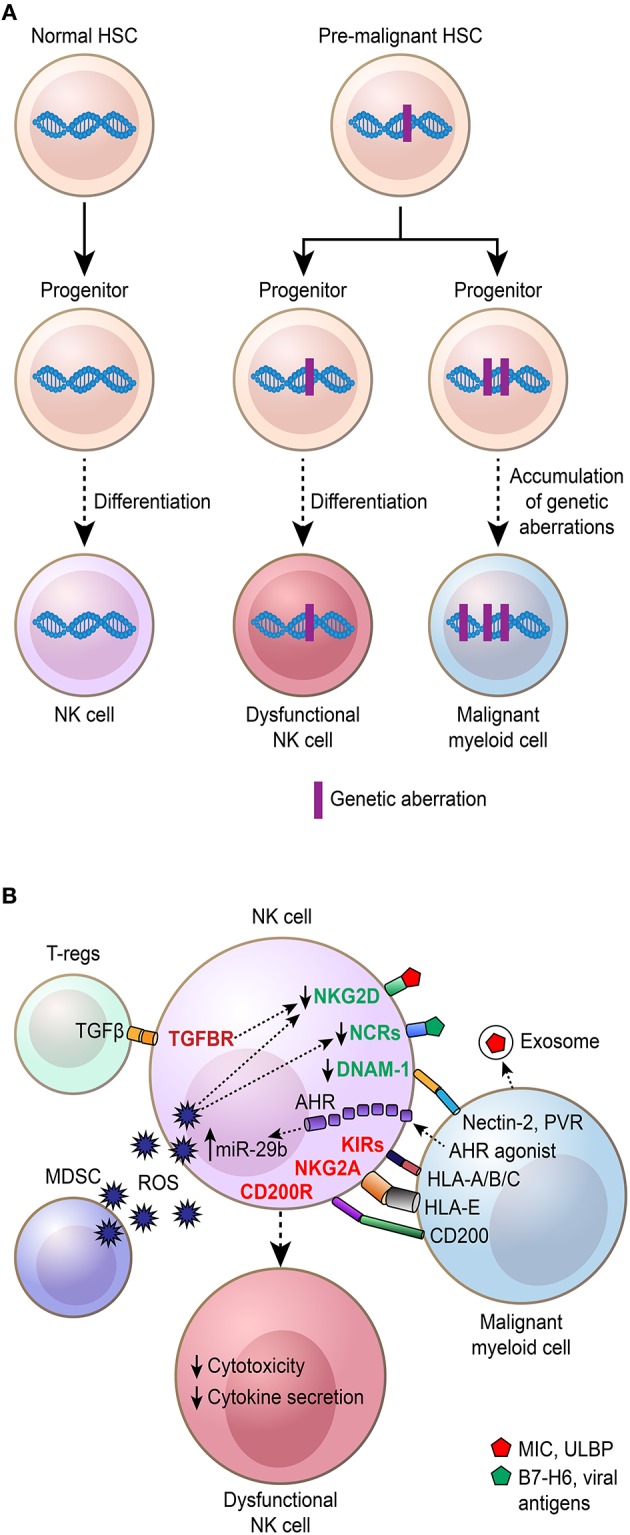
Mechanisms behind NK cell dysfunction and phenotypic/maturation alterations in myeloid malignancies. Schematic illustration showing how various mechanisms contribute to NK cell dysfunction and phenotypic/maturation alterations. **(A)** NK cells arise from hematopoietic stem cells (HSCs) that through progenitor stages differentiate to mature NK cells (purple). Initiating genetic aberrations in myeloid malignancies are thought to arise in HSCs, referred to as pre-malignant HSCs, that among other cell types give rise to NK cells, which are dysfunctional in their cytotoxic capacity and have altered maturation. **(B)** NK cells are regulated by various secreted factors and cell–cell interactions affecting their cytotoxic and cytokine-secreting capacity. Regulatory T cells (Tregs), myelo-derived suppressor cells (MDSC), and malignant myeloid cells contribute to the suppression of NK cells that become dysfunctional with altered cytokine secretion and reduced cytotoxic capacity.

The cellular origin of myeloid malignancies is thought to be a normal HSC that first acquires genetic lesions that give rise to a pre-malignant clone (105–107). In support of this hypothesis, early genetic aberrations associated with clonal hematopoiesis and myeloid malignancies can be found in multiple hematopoietic lineages, including NK cells, affecting their function (Figure 2A). Although NK cells isolated from chronic phase CML patients were found to be *BCR/ABL1* negative (108, 109), Nakajima et al. observed *BCR/ABL1*^+^ NK cells in advanced phases of the disease (110). The reason why *BCR/ABL1*^+^ NK cells are found predominantly in advanced phases of the disease is currently unclear but might be due to an expansion of the malignant stem cell pool during disease progression that gradually outcompetes normal HSCs. By contrast, T cells were always *BCR/ABL1* negative, suggesting that the presence of *BCR/ABL1* is not compatible with T cell development (110). To evaluate the impact of *BCR/ABL1* on NK cell differentiation and function, *BCR/ABL1* was introduced into cord blood CD34^+^ cells and the NK92 NK cell line (111). Enforced *BCR/ABL1* expression in cord blood CD34^+^ cells resulted in altered NK cell differentiation (110), and in NK92 cells, a decreased cytotoxicity was observed (112). Consistent with these findings, *BCR/ABL1*^+^ NK cells from CML patients grown in culture had reduced cytotoxic and proliferative capacity (113). In contrast, *BCR/ABL1*^+^ dendritic cells selectively activate NK cells, demonstrating that NK cells can also be affected by other non-myeloid cell lineages that express *BCR/ABL1* (114).

Although early studies did not detect chromosomal aberrations in NK cells from MDS patients (115, 116), later studies reported aneuploid NK cells ranging from 20 to 60% in MDS (78, 117). In addition to acquired mutations shared with the malignant cells and NK cells in patients, certain congenital mutations that pre-dispose for MDS/AML are associated with defects in NK cells. One such example is SAMD9L gain-of-function mutations that pre-disposes for MDS and are associated with defects in myeloid cells, B and NK cells (118). Also, constitutive Gata2 mutations that pre-disposes for MDS/AML are associated with alterations in NK cells as evidenced by an accumulation of terminally differentiated NK cells (119). In AML, DNMT3A mutations, which are early and often initiating events associated with clonal hematopoiesis (120), are found in NK cells, but to a lesser extent in B and T cells (121).

Taken together, early genetic aberrations driving malignant transformation are detected in a substantial fraction of NK cells in patients with myeloid malignancies. Some of these aberrations as exemplified by enforced *BCR/ABL1* expression in NK cells negatively affect NK cell cytotoxicity and differentiation. Future studies combining genetic characterization by massive parallel sequencing of NK cells with functional NK cell assays are expected to further clarify the full functional impact of cancer-associated genetic aberrations co-existing in NK cells.

## Mechanisms Suppressing NK Cells in Myeloid Malignancies and Mediating Escape From NK Cell Recognition

As appreciated from the previous section, NK cells in patients with CML, AML, and MDS are often, if not always, dysfunctional compared to healthy control NK cells. An array of mechanisms has been identified, including but not limited to soluble factors, cell-to-cell interactions, and other regulatory elements in the tumor microenvironment (Figure 2B). As described above, mutations affecting the NK cell population can also contribute to poor function of these effector cells (118). Below, we will discuss the so far known mechanisms driving the development of dysfunctional NK cells in these diseases.

Several studies published today have linked poor NK cell function with altered NK cell subset composition, phenotype, and ability to form a fully functional immunological synapse (38, 39, 122–127). In some cases, these alterations have been linked to poor clinical outcome (38, 125). Most of these studies have highlighted down-regulation of key activation NK cell receptors such as NKG2D, DNAM-1, and the NCRs, down-regulations that do not seem to correlate with the subtype of AML or MDS (38, 39, 122–124, 128). Nevertheless, studies have shown that the loss of these receptors positively correlates to the leukemia burden in the patients (38, 39, 123) and that it can be fully, or at least partially, restored in patients achieving CR following chemotherapy (38). In fact, data show that NK cell-to-tumor cell interactions can trigger the loss of DNAM-1 and NCRs (37, 38, 126, 129). Receptor–ligand interactions, triggering internalization of the activation NK cell receptor, has been highlighted as one of the most critical mechanisms (37, 38, 129, 130). Loss of activating receptors, such as NKG2D, can also be triggered by the presence of soluble molecules in the tumor microenvironment. As shown by Boissel et al. and several other groups, soluble NKG2D ligands (NKG2D-Ls) including MICA, MICB, ULBP1, and ULBP2, shedded by the tumor cells *per se*, and tumor exosomes expressing NKG2D-Ls trigger the reduction of NK cell surface NKG2D (131–135). In this context, it should be highlighted that reports indicate that AML blasts, including AML stem cells, may also evade NK cell-mediated killing by expressing low or no NKG2D-Ls (52, 136, 137). The NKG2D receptor can also be down-modulated via cytokines such as TGF-β (138). In addition to these mechanisms governing suppressed NK cell function leading to poor NK cell-mediated targeting of leukemic cells, data from a pre-clinical animal model on *de novo* AML along with collected NK cell from patients with AML have indicated that the microRNA (miRNA) miR-29b, a regulator of T-bet and Eomes, can be elevated in NK cells via AML cell-induced activation of the transcription factor aryl hydrocarbon receptor that directly up-regulates miR-29b expression resulting in incomplete maturation and poor cytotoxicity (96, 139). Other soluble mechanisms involve the release of Tim-3 that prevent the release of IL-2 while increasing the release of galectin-9 and thereby hamper NK cell cytotoxicity and targeting of primary AML cells (140). Despite that CD137 (4-1BB) is a therapeutic target for agonistic antibodies in clinical development that stimulate NK cells and T cells (141), stimulation of CD137 expressed on the surface of activated NK cells has been shown to suppress their function in AML (142). The CD137L was primarily identified in AML of the monocytic lineage, although it was found on other AML subtypes too (142). Further studies are needed to dissect the exact role for this interaction in regulating NK cell function in myeloid malignancies.

Several other mechanisms behind the escape of myeloid malignancies from NK cell recognition have also been described. Data show that down-regulation of ligands for DNAM-1 on the leukemic cell surface renders the cells resistant to NK cell targeting (143). Another study suggests that the leukemic blasts can avoid NK cell recognition by expressing low levels of NCR and NKG2D ligands, a resistance that can be reverted following exposure to differentiation-promoting myeloid growth factors and IFN-γ (136). In a separate study, expression of the oncogenic fusion proteins PML-RARA and AML1-ETO found in acute promyelocytic leukemia (APL) and some non-APL AMLs, respectively, was associated with the loss of the 2B4 ligand CD48 on the leukemia cell surface (144). Interestingly, CD48 expression was increased on APL cells following exposure to an HDAC inhibitor (HDACi). On the contrary, increased levels of IFN-γ in the tumor microenvironment may lead to up-regulation of HLA class I, and especially HLA-E, on the tumor cells leading to immune escape by inhibition of NK cells via the CD94/NKG2A receptor (145). Along these lines, up-regulation of the glycoprotein CD200 on AML cells resulted in escape from NK cell-mediated lysis via interaction with the CD200 receptor on the NK cell surface, a phenomenon that was restored using a CD200 inhibitory antibody (128).

Factors in the tumor microenvironment can also play a critical role. In addition to suppressed NK cell function, it has been demonstrated that NK cell proliferation can be inhibited by the tumor while not influencing the NK cell viability and cytotoxicity *per se* (146). As shown in CML, AML, and CMML, reactive oxygen species (ROS) can trigger both apoptosis of NK cells in the tumor microenvironment but also reduced NK cell function connected to reduced expression of activation NK cell receptors (147–149). Data also show that cell-to-cell interactions between AML cells and mesenchymal stromal cells render the AML cells less susceptible to NK cells (150). More details on the role for the tumor microenvironment learnt from other malignancies are not discussed in this review as they have been reviewed elsewhere (151).

In conclusions, an array of mechanisms has been proposed to trigger NK cell suppression, reduced NK cell numbers, and escape from NK cell-mediated recognition. Most of them have been addressed in studies on tissue samples from patients or in *ex vivo* experiments with NK cell co-cultures. Although shown in experimental animal mouse models (152), the loss of function of adoptively infused NK cells in human has not yet been systematically addressed. Nevertheless, understanding these mechanisms is key to developing new NK cell-based therapies against myeloid malignancies, especially those relying on endogenous NK cells and that may lead to long-term non-chemotherapy-based control of these diseases. The next section will discuss means to restore and/or trigger anti-leukemic responses and tumor control by NK cells.

## Means to Restore NK Cell Function and Trigger Their Cytotoxicity Against Myeloid Malignancies

**Figure 3:**
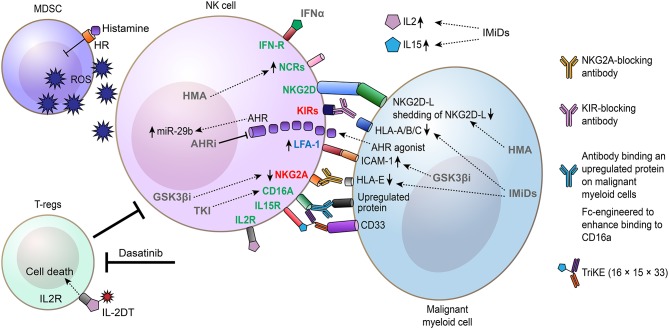
Drugs and approaches explored to restore and augment the antileukemia-capacity of NK cells. Schematic illustration showing how drugs can restore, augment, and direct NK cell-mediated killing of malignant myeloid cells. Drugs promoting NK cell-mediated killing of myeloid malignant cells by directly affecting NK cells, inhibiting regulatory T cells (Tregs) or myelo-derived suppressor cells (MDSC), and/or affecting malignant myeloid cells are shown. IL-2DT, IL-2 diphtheria toxin fusion protein. IMiDs, immunomodulatory imide drugs (include thalidomide and analogs such as lenalidomide and pomalidomide). AHRi, aryl hydrocarbon receptor inhibitor; GSK3βi, GSK3β inhibitor; HMA, hypomethylating agents (includes azacytidine and decitabine).

Dysfunctions of NK cells associated with myeloid malignancies restrain tumor immune surveillance, but may also limit therapeutic options that depend on NK cells for their mode-of-action. In addition to drugs used in the clinic that restore NK cells such as TKI for CML and hypomethylating agents for MDS and AML, a number of pharmacological strategies to re-establish and/or bolster NK cell function, including cytokines, engineered antibodies, and small-molecule drugs, are currently being explored with the aim of utilizing the endogenous NK cells to clear and control myeloid malignancies (Figure 3).

### Cytokines Including Histamine and IL-2

In 1998, high-dose IL-2 was the first immunotherapy approved for metastatic malignant melanoma and showed durable responses in a subset of patients (153). Although associated with significant toxicity, IL-2 therapy demonstrated that cytokine-induced activation of the immune system, including T cells and NK cells, can have long-term beneficial effects in certain cancers. IL-2 have in pre-clinical studies shown therapeutic efficacy by restoring NK cell receptor expression and bolster NK cell cytotoxicity against autologous AML blasts *in vitro* (48), but clinical studies evaluating IL-2 monotherapy in AML and MDS have been disappointing (154–156). However, in contrast to monotherapy, Brune et al. demonstrated that combining histamine with low-dose IL-2 treatment in AML results in improved leukemia-free survival (157). Histamine dihydrochloride acts by enhancing the immune-promoting properties of IL-2 by reducing production of immunosuppressive reactive oxygen species (ROS) (158), which leads to expansion of CD56^bright^ NK cells (90, 159). For this therapy, a high expression of NKp30 and NKp46 on CD16a^+^ NK cells before and during treatment predicted leukemia-free and overall survival (90). In addition to activating NK cells, it is a concern that IL-2 also stimulates Tregs, which are immunosuppressive and counteract NK cell activation (160). For histamine with low-dose IL-2 treatment, a promising observation was that the increase in Tregs was transient, whereas the increase in NK cells was more long-lasting (161). In contrast to IL-2, IL-15 that activates memory T cells and bulk NK cells is associated with less toxicity, suggesting that IL-15 has several advantages over IL-2 in a clinical setting (160). Partially, by inducing the expression of the activating NK cell receptor NKp30, IL-15 was found to enhance the cytotoxicity of NK cells from AML patients (162, 163). When expressed in a non-secreted form in NK cells, IL-15 stimulated autonomous NK cell growth and increased their cytotoxicity against leukemia and lymphoma cells in cultures and in mice (164). However, recent reports indicate that chronic or repetitive exposure of IL-15 to NK cells lead to NK cell exhaustion (165, 166), suggesting that the long-term effects of IL-15 should be carefully monitored in future studies.

In CML, interferon alpha (IFN-α) was used as a standard treatment prior to the TKI era. Although the full mechanistic basis for how IFN-α has antileukemic activity is unknown, IFN-α has been shown to boost the function of endogenous NK cells (167). Another cytokine that has been shown to bolster NK cells in CML is IL-2. In line with findings described above for IL-2 in AML, Cervantes et al. used IL-2 to stimulate autologous NK cells and demonstrated selective suppression of CML progenitor cells relative to corresponding normal progenitors (47).

### Small-Molecule Drugs

As discussed in section Mechanisms Suppressing NK Cells in Myeloid Malignancies and Mediating Escape From NK Cell Recognition, one mechanism that has been put forward to explain impaired tumor immune surveillance by NK cells in myeloid malignancies is long-term exposure of soluble NKG2D ligands such as MICA, MICB, and ULBP2 secreted by the malignant blasts. Consistent with this notion, hypomethylating agents (azacytidine and decitabine) that are used to treat AML and MDS patients were found to decrease shedding of MICA, MICB, and ULBP2 and restore NK cell function (168). In line with these findings, Vasu et al. reported that decitabine enhances NK cell cytotoxicity induced by an anti-CD33 monoclonal antibody (mAb) against AML blasts associated with up-regulation of NKG2D (169). In an AML xenograft mouse model, decitabine treatment potentiated NK cell-mediated killing of the AML cells by NKp44 up-regulation, suggesting that hypomethylating agents are promising drugs for enhancing NK cell activity by multiple mechanisms (170). Complementary to decitabine, which up-regulates NKG2D, the HDACi valproic acid was found to induce the expression of NKG2D-Ls on AML cells, rendering them more sensitive to lysis by NK cells (171). Another approach to enhance NK cell function in AML is inhibition of glycogen synthase 3 kinase beta (GSK3β). Parameswaran et al. provided pharmacological and genetic evidence that inactivation of GSK3β restores NK cells from AML patients resulting in enhanced killing of autologous leukemic cells (172). Mechanistically, GSK3β inhibition promoted up-regulation of LFA-1 on NK cells and its partner ICAM-1 on AML cells, associated with increased AML–NK cell conjugates (172).

Another clinically approved drug that improves NK cell function is lenalidomide, used for treatment of multiple myeloma, 5q- MDS, and B-cell lymphomas (173). In patients with relapsed/refractory solid tumors or MDS, lenalidomide treatment was found to increase IL-2 and IL-15 levels accompanied by restoration of NK cell function (174). Similar to AML, lenalidomide and its derivate pomalidomide potentiated NK cell function (175). The antileukemic activity of these drugs was associated with down-regulation of HLA class I molecules on the AML cells (175). Although lenalidomide has been shown to achieve anti-cancer activity by inducing degradation of essential proteins for 5q- MDS and multiple myeloma cells (176, 177), the mechanistic basis for how lenalidomide activates NK cells is currently unclear.

In CML, a somewhat unexpected finding is that several TKIs (imatinib, dasatinib, and nilotinib), dasatinib in particular, induces expansion of NK cells from diagnostic values, indicating that these therapies promote tumor immune surveillance mediated by NK cells (178, 179). Moreover, TKI therapy results in improved NK cell function and killing of leukemic cells (180). Recent findings revealed that the restored NK cell function by dasatinib treatment is coupled to down-regulation of the NK cell inhibitory receptor NKG2A (181). A positive effect of dasatinib on the immune system was suggested to persist even long-term after stopping treatment, as a CML patient remained in MR several years post-treatment, associated with cellular immunity by memory and effector cytotoxic T lymphocytes and NK cells (182).

### Antibody-Based Therapies That Depend on NK Cells for Eradicating Myeloid Malignancies

Therapeutic antibodies can achieve anti-tumor responses not only by modulating the activity of their protein targets but also by redirecting effector cells of the immune system to the cancer cells. By targeting cell surface proteins up-regulated on the malignant cells, a selective immune response can be activated against the cancer cells. In particular, NK cells are critical effector cells for eliciting ADCC. Therapeutic antibodies designed to induce ADCC are predominantly of IgG1 isotype and bind to an antigen on cancer cells and to the low-affinity CD16a on NK cells with their Fc domain. In addition to physically linking the malignant cells and NK cells together, binding of the antibody to CD16a is sufficient to activate the NK cells and induce ADCC, even without additional activation signals (12, 15). One such example is Rituximab, which targets CD20 on B cells, and is used today for treatment of several forms of B cell malignancies (183). Consistent with NK cells playing a key role in mediating ADCC upon Rituximab treatment, patients homozygous for the single-nucleotide polymorphism CD16a-158V, which bind IgG1 with higher affinity than CD16a-158F, showed improved clinical response to Rituximab (184, 185).

For myeloid malignancies, there is a strong rationale to target a chemotherapy-resistant reservoir of self-renewing leukemia cells, referred to as leukemia stem cells, as these are associated with disease relapse after initial responses to therapy (107, 186, 187). Consistent with this hypothesis, antibodies directed to IL3Rα (CD123), which is up-regulated on AML stem-cell-enriched cells, showed anti-leukemic activity in pre-clinical models of AML (188, 189). To enhance the binding to CD16a, an Fc-engineered anti-CD123 antibody was developed that showed superior NK-cell mediated killing of leukemia stem cells in AML and CML (190–192). Similarly, an antibody that binds to CD133 on myeloid cells and with amino acid substitutions (S293D/I1332E) in the Fc domain for enhanced binding to CD16a induced strong degranulation and lysis of CD133-expressing AML cells in the presence of either autologous or allogeneic NK cells (193). Interleukin 1 receptor accessory protein (IL1RAP) is another candidate therapeutic target up-regulated on leukemia stem cells in myeloid malignancies (194–196). Consistent with IL1RAP being up-regulated on leukemia stem cells vs. normal hematopoietic stem and progenitor cells, IL1RAP-targeting antibodies with enhanced CD16a-binding capacity induced selective NK cell-mediated ADCC when exposed to candidate leukemia stem cells (196). Moreover, Ågerstam et al. demonstrated that IL1RAP-targeting antibodies exhibited potent antileukemic efficacy in CML and AML xenograft models (197, 198).

Another promising approach to direct the immune system to kill cancer cells is the use of bispecific antibody-based modalities that can be designed to bind one antigen on the cancer cell and a separate antigen on a cytotoxic immune cell. By using a Bispecific Killer Engager (BiKE) consisting of a single-chain variable fragment (scFv) targeting CD16a on NK cells and a scFv targeting CD33 on AML cells, NK cell-mediated cytotoxicity and cytokine release could be effectively triggered (199). With the aim to boost NK cell activity and persistence, as a further development of the 16 × 33 BiKE targeting CD16a and CD33, IL-15 Trispecific Killer Engagers (TriKE) referred to as 16 × 15 × 33 TriKEs have been developed (200). When compared to the 16 × 33 BiKE, Vallera et al. demonstrated that the 16 × 15 × 33 TriKE induced superior NK cell cytotoxicity and cytokine release when exposed to AML cells (200).

### Prevent Suppression From the Microenvironment

Certain types of immune cells are immune suppressive and can restrain immune-mediated attacks against malignant cells. Both Tregs and myeloid-derived suppressor cells (MDSCs) have been shown to restrain NK cells, hence, therapeutic interventions aimed at depleting either of these cells have the potential to enhance NK cell activity (201). One approach to deplete MDSCs is the use of the 16 × 15 × 33 TriKEs, which, in addition to killing CD33^+^ malignant cells, are also effective in killing CD33^+^ MDSCs, leading to restoration of NK cell function in MDS (202, 203). In CML patients, dasatinib treatment is associated with inhibition of Tregs. Consistent with this hypothesis, the response rate after 18 months' treatment with dasatinib was significantly better in CML patients with low numbers of Tregs that inversely correlated with NK cell counts, indicating that inhibition of Tregs by dasatinib enhances NK cell-mediated killing of leukemic cells (102). The TNF family member receptor activator for NF-KB ligand (RANKL) is mainly known as a regulator of bone remodeling but also regulates immune functions. Activation of RANKL signaling in AML cells result in secretion of immune-modulatory factors that impaired NK cell function (204). Consistent with this finding, treatment of AML cells with Denosumab, an inhibitory RANKL antibody, resulted in enhanced NK cell function (204).

### Checkpoint Inhibition

Immune checkpoint inhibitors targeting the PD1/PDL1 interaction have been clinically validated and show remarkable response rates in several forms of cancer. Mechanistically, the selective anti-tumor effect of the T cells is based on the recognition of tumor neo-antigens presented on HLA class I molecules. With a high mutational burden in certain cancers, more tumor neo-antigens are formed and recognized by the T cell receptors. As myeloid malignancies have a relatively low mutational burden, immune checkpoint inhibitors for T cells are expected to be less effective in disorders such as AML. However, recent data by Hsu et al. proposed that NK cells express PD1 and that blockade of the PD1/PDL1 interaction also activates NK cells that are indispensable for the therapeutic effect of these therapies (205). Hence, blocking PD1/PDL1 may show unexpected therapeutic efficacy in myeloid malignancies by activating NK cells, possibly in combinations with other therapies, a route that warrants further investigations.

As postulated by the “missing-self” hypothesis (9), NK cells are regulated by inhibitory HLA class I molecules that bind to their cognate KIRs on NK cells. To enhance NK cell activity, the mAb 1-7F9 that cross-reacts with KIR2D molecules and block the interaction with virtually all HLA-C molecules was developed (206). In the presence of NK cells, 1-7F9 induces selective killing of HLA-C expressing AML cells vs. normal peripheral blood mononuclear cells (206). When evaluated in a phase I study in AML, increased expression of the activation marker CD69 on NK cells was observed and relapse-free survival compared favorable to historical data from comparable patient cohorts (207). Blocking KIRs also augments ADCC induced by antibodies binding to CD20 and CD33, suggesting that KIR blockade can enhance the efficacy of therapeutic antibodies that rely on ADCC for killing of cancer cells (206, 208). However, based on data claiming that the anti-KIR antibody can rapidly detune NK cell function *in vitro* and in cancer patients (209), thereby limiting its therapeutic efficacy, and given the pre-clinical data indicating that KIR blockade augments ADCC (206, 208), better responses are likely to be achieved when combining KIR blockade with other drugs that boost NK cell cytotoxicity. In addition to tumor-targeting antibodies, drugs such as lenalidomide that is reported to boost NK cell function *per se*, and maybe also decitabine or HDACi as discussed above, may be relevant. Further studies are needed to fully delineate the efficacy of such approaches and if it induces durable remissions. In addition to KIR, a subset of NK cells expresses the inhibitory receptor NKG2A that bind to HLA-E on healthy and cancer cells. In line with a key role for NKG2A in immune checkpoint regulation, Ruggeri et al. demonstrated that targeting of NKG2A with a blocking antibody resulted in strong NK-cell mediated anti-leukemic activity in mice engrafted with primary leukemia cells (210). Similar data for KIR and NKG2A have also been generated in *ex vivo* experiments by others (53). Again, it should be highlighted that targeting these receptors alone may have limited efficacy due to the risk of detuning of baseline NK cell cytotoxicity and that combination therapies may generate better results.

In summary, several clinically approved drugs and drugs in pre-clinical development can be utilized to improve NK cell function by distinct mechanisms. Hence, identifying beneficial combinations of these therapies in a disease- and genotype-specific manner has the potential to not only restore tumor immune surveillance in patients with myeloid malignancies, but also further enhance NK cell activity over normal baseline levels. If further combined with other immunotherapies or targeted therapies that neutralize oncogenic drivers, multiple therapies can be used simultaneously to attack the malignant cells, a strategy that will minimize the risk for resistance mechanisms to arise and may ultimately lead to cure of patients.

## Concluding Remarks and Future Outlook

In recent years, significant advances have been made in our understanding of the role for NK cells in myeloid malignancies. We have become aware of the idea that NK cells in patients with MDS, AML, and CML most often are dysfunctional, but also that their phenotype and function can be partially restored following administration of tumor-targeting drugs such as TKI, chemotherapy, and hypomethylating agents, and also by immunostimulatory agents such as cytokine-based therapies. Data also demonstrate that such restoration of the endogenous NK cell function can be key in achieving durable responses in subgroups of patients. Although therapeutic strategies involving adoptive NK cell infusions hold promise, with objective clinical response rates of 30–50% in patients with advanced disease such as relapsed and/or refractory AML and high-risk MDS, these results are only transient and non-curative today. Therefore, a tempting and, in many ways, more natural approach to achieve long-term remissions would be to redirect the endogenous NK cells to target and control the disease. This notion is based on the ample support for NK cell-mediated immunosurveillance of myeloid malignancies along with the abovementioned data demonstrating that endogenous NK cells can be key to attain durable remissions, a phenomenon that is in line with that observed for donor NK cells in preventing leukemia relapse in certain settings of allogeneic SCT. Identifying therapies that redirect endogenous NK cells is especially of interest given the aging population, in which more and more patients are ineligible for an allogeneic SCT or even to high-intensity chemotherapy. In addition to its simplicity and potential to serve the broader population, the current high costs for SCT and cellular immunotherapies favor this alternative approach.

However, to be able to develop effective therapies that rely on endogenous NK cells, we need to better understand what factors that cause tumor evasion from NK cells and identify drugs that prevent or neutralize them. We also need to recognize what drugs can be utilized to selectively augment the tumor killing capacity of the endogenous NK cells *per se* and the temporal aspects of using these drugs. Moreover, to achieve durable disease control, we need to identify therapies that not only activate the NK cells short-term and potentially exhaust them, but more importantly to develop drugs or approaches/protocols that stimulate the NK cells for enhanced tumor immune surveillance long-term. To this end, combinational therapies and/or sequential therapies may be required for achieving significant clinical responses. Nevertheless, it will be critical to, in more detail, understand the processes that govern NK cell development and how it is perturbed in disease, findings that may translate into new therapeutic opportunities. Lessons may also be learnt from studies on ALL, as this leukemia seems to be less vulnerable to targeting by NK cells compared to AML. Such studies could potentially improve our understanding of the molecular specificity of NK cell killing of leukemic cells in general but also evasion mechanisms employed by the ALL cells *per se* as well as factors in the bone marrow environment of that disease in particular. As mentioned in this review, both cytokines, antitumor antibodies, including BiKEs, and TriKEs, and checkpoint inhibitors hold promise for the treatment of myeloid malignancies but need to be studied in greater detail until their full potential can be expected. We also need to identify new molecules to target in order to explore new therapeutic opportunities as well as biomarkers to monitor NK cell function during treatment. While this is explored, we will likely start receiving the first insights into the potential role for CAR-NK cells in treating cancer, which hopefully will contribute to our understanding while adding another layer of immunological pressure to retain the myeloid malignancy in remission. Compared to CAR-T cells that can induce toxic and even lethal cytokine release syndromes and neurotoxicity, the CAR-NK cells are expected to be better tolerated, but their potential short persistence in patients might limit their clinical use. Several molecular targets expressed on myeloid leukemia cells, such as CD123 and CD33 but also NKG2DLs and CD7, are currently being explored in the CAR field and more efficient protocols for CAR-NK cell development are being established. However, the discovery of additional and potentially more suitable molecular targets is needed to more selectively target the malignant myeloid cells while sparing normal cells. Another important aspect is also that the suppressed autologous NK cells in myeloid malignancies used for reprogramming to CAR-NK cells need to have restored or ideally enhanced function prior to reprogramming and that mechanisms potentially dysregulating the CAR-NK cells following re-infusion need to be controlled. This also applies if using IPS- or cord blood-derived CAR-NK cells. Hence, drugs and approaches discussed in this review are utterly important and need further attention also in relation to CAR-NK cells against myeloid malignancies to induce and maintain durable remissions.

Based on the data presented in this review, we strongly believe that new unique opportunities to better utilize NK cells to induce long-term remissions in patients with myeloid malignancies will be a reality in the near future.

## Author Contributions

MC and MJ have contributed equally in the outline and writing of the manuscript as well as for the design of the figures.

### Conflict of Interest

The authors declare that the research was conducted in the absence of any commercial or financial relationships that could be construed as a potential conflict of interest.
